# Genome Sequence of an Arthroconidial Yeast, Saprochaete fungicola CBS 625.85

**DOI:** 10.1128/MRA.00092-19

**Published:** 2019-04-11

**Authors:** Broňa Brejová, Hana Lichancová, Viktória Hodorová, Martina Neboháčová, Ľubomír Tomáška, Tomáš Vinař, Jozef Nosek

**Affiliations:** a Department of Computer Science, Faculty of Mathematics, Physics and Informatics, Comenius University in Bratislava, Mlynská dolina, Bratislava, Slovak Republic; b Department of Biochemistry, Faculty of Natural Sciences, Comenius University in Bratislava, Ilkovičova, Bratislava, Slovak Republic; c Department of Genetics, Faculty of Natural Sciences, Comenius University in Bratislava, Ilkovičova, Bratislava, Slovak Republic; d Department of Applied Informatics, Faculty of Mathematics, Physics and Informatics, Comenius University in Bratislava, Mlynská dolina, Bratislava, Slovak Republic; University of California, Riverside

## Abstract

Saprochaete fungicola is an arthroconidial yeast classified in the Magnusiomyces/Saprochaete clade of the subphylum Saccharomycotina. Here, we report the genome sequence of holotype strain CBS 625.85, assembled to five putative chromosomes. The genome sequence is 20.2 Mbp long and codes for 6,138 predicted proteins.

## Abstract (precis)

Saprochaete fungicola is an arthroconidial yeast classified in the Magnusiomyces/Saprochaete clade of the subphylum Saccharomycotina. Here, we report the genome sequence of holotype strain CBS 625.85, assembled to five putative chromosomes.

## ANNOUNCEMENT

*Saprochaete fungicola* is an anamorphic yeast reproducing by fragmentation of hyphae into asexual spores dubbed arthroconidia (1). Arthoconidia facilitate dissemination, and in some pathogenic fungi, their formation contributes to virulence (2). To provide a resource to study genetic control of arthroconidiogenesis, we determined the genome sequence of the strain CBS 625.85, originally isolated from ascocarps of Nectria cinnabarina, a plant pathogenic fungus causing coral spots (1). Genomic DNA sequencing was performed using a combination of HiSeq 2000 (Illumina) and MinION (Oxford Nanopore Technologies) platforms. DNA was isolated using a standard protocol (3), and total cellular RNA was prepared from a culture grown in yeast extract-peptone-galactose (YPGal) medium (1% [wt/vol] yeast extract, 2% [wt/vol] peptone, and 2% [wt/vol] galactose) at 28°C using hot phenol extraction (4) and an RNeasy minikit (Qiagen).

In total, 3.0 Gbp (∼148× genome coverage) were sequenced in 309,350 long reads (mean, 9.7 kbp; longest read, 251 kbp) using a MinION Mk-1B device with an R9.4.1 flow cell and SQK-LSK109 kit. The paired-end (2 × 101 nucleotides [nt]) TruSeq PCR-free DNA library was sequenced on a HiSeq 2000 instrument at Macrogen Korea, yielding 40,313,550 short reads (4.1 Gbp; coverage, ∼203×). Finally, 38,086,316 transcriptome sequencing (RNA-Seq) reads were generated from a TruSeq nonstranded mRNA paired-end (2 × 101 nt) library using a NovaSeq 6000 instrument at Macrogen Korea.

Assembly with SPAdes v. 3.12.0 (5) resulted in 367 contigs (*N*_50_ value, 0.4 Mbp), and long-read assembly with miniasm v. 0.3-r179 (6) and minimap2 v. 2.13-r852 (7) polished by racon v. 1.3.1 (8) yielded 10 contigs (*N*_50_ value, 5.1 Mbp). Two contigs contained mitochondrial DNA (mtDNA), of which one complete copy (circular 33-kbp contig) was retained for the final assembly. Two contigs not supported by Illumina reads were discarded as contamination. One ribosomal DNA (rDNA) contig was discarded, and eight copies of the rDNA cluster were retained elsewhere. To further correct the long-read assembly, the rDNA cluster was polished separately by two iterations of pilon v. 1.21 (9) with BWA-MEM v. 0.7.17-r1188 (10), and four contig ends were extended using SPAdes contigs. Identified by a significant decrease in long-read coverage, five local misassemblies were corrected, also using SPAdes assembly. All changes were based on reliable long-range overlaps and supported by multiple long reads. The final assembly contains five nuclear contigs of lengths 5.4, 5.1, 4.4, 3.2, and 2.1 Mbp, with an overall G+C content of 40.6%. These likely correspond to full-length chromosomes since they terminate on both ends by putative telomeric repeats ([AACAG]_0–1_A_2–6_G_0–1_A_0–1_G_4–7_) with the predominating motif A_2_GAG_6_.

RNA-Seq reads, processed by Trimmomatic v. 0.36 (11), were assembled into transcripts by Trinity v. 2.8.4 (12) and aligned to the genome by blat v. 36 × 2 (13). Augustus v. 3.2.3 (14) trained on the related Magnusiomyces capitatus genome (15) with RNA-Seq evidence was used for initial gene predictions. Of these genes, 4,785 best supported by RNA-Seq transcripts (99% identity on 99% of length as identified by blat) were used to retrain Augustus parameters for *S. fungicola* and, together with the RNA-Seq evidence, to predict the final set of 6,138 protein-coding genes. The high-contiguity genome sequence of *S. fungicola* will be instrumental in comparative and functional studies focused on biology and evolution of arthroconidial yeasts.

### Data availability.

The genome assembly has been deposited in ENA under the accession number CAACAH010000000, and the Illumina, MinION, and RNA-Seq reads were deposited in SRA under the accession numbers ERR3046939, ERR3046967, and ERR3046965, respectively. The genome annotations are available through a genome browser at http://genome.compbio.fmph.uniba.sk/ and are also archived through Zenodo (16).
